# Lipid Profile and Atrial Fibrillation: Is There Any Link?

**DOI:** 10.31083/j.rcm2308272

**Published:** 2022-07-26

**Authors:** Qi Jiang, Ling Yang, Ming-Long Chen, Fei Hua, Jian-Jun Li

**Affiliations:** ^1^Department of Cardiology, The Third Affiliated Hospital of Soochow University, 213003 Changzhou, Jiangsu, China; ^2^Department of Cardiology, The First Affiliated Hospital of Nanjing Medical University, 210029 Nanjing, Jiangsu, China; ^3^Department of Endocrinology, The Third Affiliated Hospital of Soochow University, 213003 Changzhou, Jiangsu, China; ^4^Cardiometabolic Center, Fu Wai Hospital, Chinese Academy of Medical Sciences and Peking Union Medical College, 100037 Beijing, China

**Keywords:** arrhythmias, atrial fibrillation, dyslipidemia, lipid profile

## Abstract

Atrial fibrillation (AF) is the most common type of symptomatic arrhythmias, which was induced by multiple causes and dyslipidemia is a well-known causal factor for the atherosclerotic cardiovascular disease (ASCVD). Interestingly, emerging data has suggested that lipid disorder may be also associated with AF. Several previous studies have shown a link of the prevalence of AF with decreased concentration of low-density lipoproteins (LDL)-cholesterol, total cholesterol (TC), high-density lipoproteins (HDL)-cholesterol, and elevated lipoprotein(a) [Lp(a)]. In this manuscript, we try to summarize the current evidence regarding the relation of dyslipidemia to the incident AF, present the potential lipid-related mechanisms of AF development, which is involved in cell membrane properties, LDL-receptors reduction, reverse cholesterol transport, adiposity-induced inflammation, apoptosis, and autophagy. Such information may boost our understandings concerning the lipid disorder and AF, which may help future exploration in the link of dyslipidemia and AF.

## 1. Introduction

Lipids such as cholesterol and triglycerides (TGs), comprised diverse classes of biomolecules, are known to play crucial roles in cell membranes, energy sources, and signaling activation [1]. Cholesterol and triglycerides require the presence of lipoproteins that assist the transport of lipids between the tissues, consisting of apolar lipid triglycerides and cholesterol esters [2]. According to the classification criteria, lipoproteins consist of chylomicrons (CM), very low-density lipoproteins (VLDL), low density lipoproteins (LDL), intermediate density lipoproteins (IDL), high-density lipoproteins (HDL), and lipoprotein(a) [Lp(a)]. Untreated total cholesterol (TC) <200 mg/dL has been defined as achieving optimal levels of a cardiovascular disease risk factor and is related to increased cardiovascular disease (CVD) and mortality [3].

To our knowledge, CVD has become a global threat to the population’s health [4]. Multiple lipid components, as we know, are related to atherosclerotic CVD, especially coronary heart disease (CHD), as the main cause of universal morbidity and mortality [5]. Atypical plasma lipid level is one of the dependent risk factors in CHD [6]. Given that patients with CHD develop primary cardiovascular events, at a rate of 20% for more than 5 years, secondary prevention from lipid profile management is critical [7]. Lipid reduction can lower the risk of cardiovascular events via the evidence obtained from genome research, mendelian randomization, and population-based observation and intervention study [8, 9, 10]. Recent studies on dyslipidemia have revealed the certain associations with other disorders, such as aortic valvular disease [11], Alzheimer disease [12], diabetes mellitus [13], and cerebral hemorrhage [14].

Atrial fibrillation (AF) is the most common symptomatic arrhythmias worldwide, and its prevalence is expected to more than double in the next 3 decades [15]. Regulating modifiable risk factors for the occurrence and progression of AF is the mainstream in current research. The correlation between plasma lipid and multiple cardiovascular disease has already been acknowledged generally. As we know, what evidence there is tends to show a definite link between higher levels of cholesterol and increased cardiovascular events. Interestingly, several recent data have examined the relationship of lipid disorder to AF and while the results are controversial, even so there is a phenomenon named as “cholesterol paradox” in AF persistence [16]. In this review, hence, we try to review previous studies pertaining to association of lipid profiles with AF in order to boost our understanding in this unique field. This review, aimed to evaluate the guiding effect on the treatment and prevention of AF from the perspective of lipid lowering.

## 2. Potential Mechanisms for Atrial Fibrillation

AF is featured by fast-frequency activation of the atria, resulting in desynchrony of atrial contraction and abnormity of ventricular activation [17]. AF may occur in the comorbid conditions, which cause structural and histopathologic changes and formed AF substrate [18], suggesting electrophysiological, mechanical, and anatomical features of the atrium. Rapid triggering has been proven to initiate propagating reentrant waves in atrial substrate via altering ion channel function [19]. Remodeling also leads to changes in calcium ion handling, which promote triggered activity and re-entry [20]. As with triggers, the vulnerable atrial substrate plays a role in AF initiation. Structural and electrophysiological atrial irregularities promote AF maintenance by stabilizing reentry [17]. The intrinsic activities of sympathetic and parasympathetic plexuses are independent of extrinsic input, contributing to AF initiation and maintenance [21]. The structural heart disease, extrinsic modulating factors, and genetic factors can induce electrical consequences. Fibrosis, a form of structural remodeling, develops AF subsequently.

The vulnerable atrial substrate was affected by comorbid conditions, genetics, sex, and other variables. The characterization of the vulnerable atrial substrate was regarded as one of the AF risk factors (RFs). AF RFs alter atrial substrate, inducing histopathologic and structural changes to atrial fibrosis. Unmodifiable RFs for developing AF include genetics, age, sex, and race [17]. AF can result in the progress of modifiable RFs, consisting of physical activity, obesity, smoking, diabetes mellitus, high blood pressure, and obstructive sleep apnea [17]. Finally, AF is related to the raised risks of stroke [22], extracranial systemic thromboembolism [23], dementia [24], heart failure [25], myocardial infarction [26], venous thromboembolism [27], and mortality [28].

## 3. Lipid Subtypes Disorders and AF

### 3.1 LDL-C and TC

#### 3.1.1 Epidemiology

Previous studies revealed that hypercholesterolemia was negatively correlated to AF, and elevated LDL-C and TC levels were related to a lower incidence rate of AF (Table 1, Ref. [29, 30, 31, 32, 33, 34, 35, 36, 37, 38]). A large cross-sectional study of 13,724 patients showed a negative relationship between AF and LDL-C (Adjusted hazard ratio [HR] (95% confidence interval [CI]) 0.60 (0.48, 0.75); *p *< 0.001), and TC (0.61 (0.49, 0.75); *p *< 0.001) [29]. Higher levels of TC (HR 0.60, 95% CI 0.43–0.84) and LDL-C (HR 0.60, 95% CI 0.43–0.83) had a negative relationship with AF in the Chinese population [30].

**Table 1.** **Atypical plasma lipid profile in the prevalence of atrial fibrillation**.

Exposure	References	Year	Population	Age (years)	Enrollment dates	OR (95% CI)	Results
LDL-cholesterol	Harrison *et al*. [29]	2020	13,724	58	2015–2016	0.60 (0.48–0.75)	High LDL-C associated with lower risk of AF prevalence
	Li *et al*. [30]	2018	88,785	50.8	2006–2007	0.60 (0.43–0.83)	High LDL-C was inversely associated with incident AF
	Xue *et al*. [31]	2019	985	63.4	2014–2017	0.56 (0.31–1.00)	Inverse association of LDL-C with new-onset AF
	Yao *et al*. [32]	2020	42,825	18–96	1997–2019	0.95 (0.92–0.97)	LDL-C inversely associated with new-onset AF
Total cholesterol	Harrison *et al*. [29]	2020	13,724	58	2015–2016	0.61 (0.49–0.75)	High TC associated with lower risk of AF prevalence
	Li *et al*. [30]	2018	88,785	50.8	2006–2007	0.60 (0.43–0.84)	High TC was inversely associated with incident AF
	Xue *et al*. [31]	2019	985	63.4	2014–2017	0.54 (0.32–0.90)	Inverse association of TC with new-onset AF
	Yao *et al*. [32]	2020	42,825	18–96	1997–2019	0.95 (0.93–0.96)	TC inversely associated with new-onset AF
HDL-cholesterol	Harrison *et al*. [29]	2020	13,724	58	2015–2016	0.58 (0.46–0.74)	AF was inversely associated with HDL-C
	Boudi *et al*. [33]	2020	6,881	67	2000–2003	0.27 (0.21–0.35)	Low HDL was the strongest predictor for AF
	Guan *et al*. [35]	2020	231,393	45.9–73.0	2005–2019	0.86 (0.76–0.97)	Elevated HDL-C levels reduced the risk of AF
LDL-C/HDL-C ratio	Harrison *et al*. [29]	2020	13,724	58	2015–2016	0.75 (0.61–0.94)	Higher LDL-C/HDL-C ratio reduced AF risk in elder≥75
	Alonso *et al*. [34]	2014	7,142	45.0–84.0	2000–2002	0.64 (0.48–0.87)	High HDL was associated with lower AF risk
Triglycerides	Harrison *et al*. [29]	2020	13,724	58	2015–2016	1.21 (0.98–1.50)	No significant difference in AF and TG levels
	Guan *et al*. [35]	2020	231,393	45.9–73.0	2005–2019	1.02 (0.90–1.17)	No significant association between TG and incident AF
	Alonso *et al*. [34]	2014	7,142	45.0–84.0	2000–2002	1.60 (1.25–2.05)	High TG was associated with higher risk of AF
Lipoprotein-a	Aronis *et al*. [36]	2017	15,792	45.0–64.0	1996–1998	0.98 (0.82–1.17)	Lp(a) was not associated with incident AF
	Garg *et al*. [37]	2020	6,814	45.0–84.0	2000–2002	0.84 (0.71–0.99)	High Lp(a) reduced the risk of incident AF
	Arnold *et al*. [38]	2021	1,759	74.4	2014–2017	0.89 (0.35–2.28)	No significant association between AF and Lp(a)

AF, atrial fibrillation; OR, odds ratio; CI, confidence interval; LDL, low density lipoproteins; TC, total cholesterol; HDL, high-density lipoproteins; TG, triglycerides; Lp(a), lipoprotein(a).

Among the 985 patients with acute ST-segment elevation myocardial infarction, inverse associations of TC (HR 0.54, 95% CI 0.32–0.90) and LDL-C (HR 0.56, 95% CI 0.31–1.00) with new-onset AF was observed [31]. Plasma levels of LDL-C and TC were negatively associated with new-onset AF while in hospital, suggesting a poor prognosis of post-discharge. LDL-C can be performed to evaluate stroke stratification in AF patients and were associated with a higher prevalence of ischemic stroke (adjusted odds ratio [OR] 2.004, 95% CI 1.624–2.473; *p *< 0.001) [39]. A meta-analysis of consolidated data from 16 studies revealed that TC and LDL-C were negatively correlated to the risk of incident AF (risk ratio [RR] 0.95, 95% CI 0.93–0.96, $I^{2}$ = 74.6%, n = 13; RR 0.95, 95% CI 0.92–0.97, $I^{2}$ = 71.5%, n = 10, respectively) [32], suggesting that higher TC and LDL-C levels were related to a lower incidence risk of AF.

The degradation progress of the LDL protein apolipoprotein B100 (apoB100) was induced by LDL oxidation. As the native or malonaldehyde-modified peptide, apoB100 peptide 210 (p210) is known as extremely immune recognized epitopes. In the Malmö Diet and Cancer cohort study, compared with the first quartile of IgM against p210, females with the fourth quartile of IgM against native p210 had a lower risk of the development of AF (adjusted HR 0.67, 95% CI 0.49–0.91, *p* = 0.01) [40].

Statin therapy has been used to reduce the concentration of LDL-C levels [41]. A population-based cohort study was also performed to evaluate the association between the use of statins and risk of long-standing persistent AF [42], consisting of 1317 patients with incident AF during the follow-up period. Compared with control, a 23% lower risk of AF was observed in statin use group. In the dose-effect relationship, the high and medium dose use of statins has a significant negative effect on the incident risk of permanent AF, except for the low-dose use group [42]. Consistent with this finding, prior meta-analyses showed that statin medication could reduce the recurrent rate of AF [43, 44, 45]. A meta-analysis of six interventional studies among 515 statin users with persistent AF was implemented to estimate the recurrent AF after electrical cardioversion [46]. The 34% risk reduction of AF recurrence after electrical cardioversion was found in patients with statin treatment, including atorvastatin (10 to 80 mg/day), rosuvastatin (20 mg/day), and pravastatin (40 mg/day).

#### 3.1.2 Pathophysiology

**Fig. 1.**
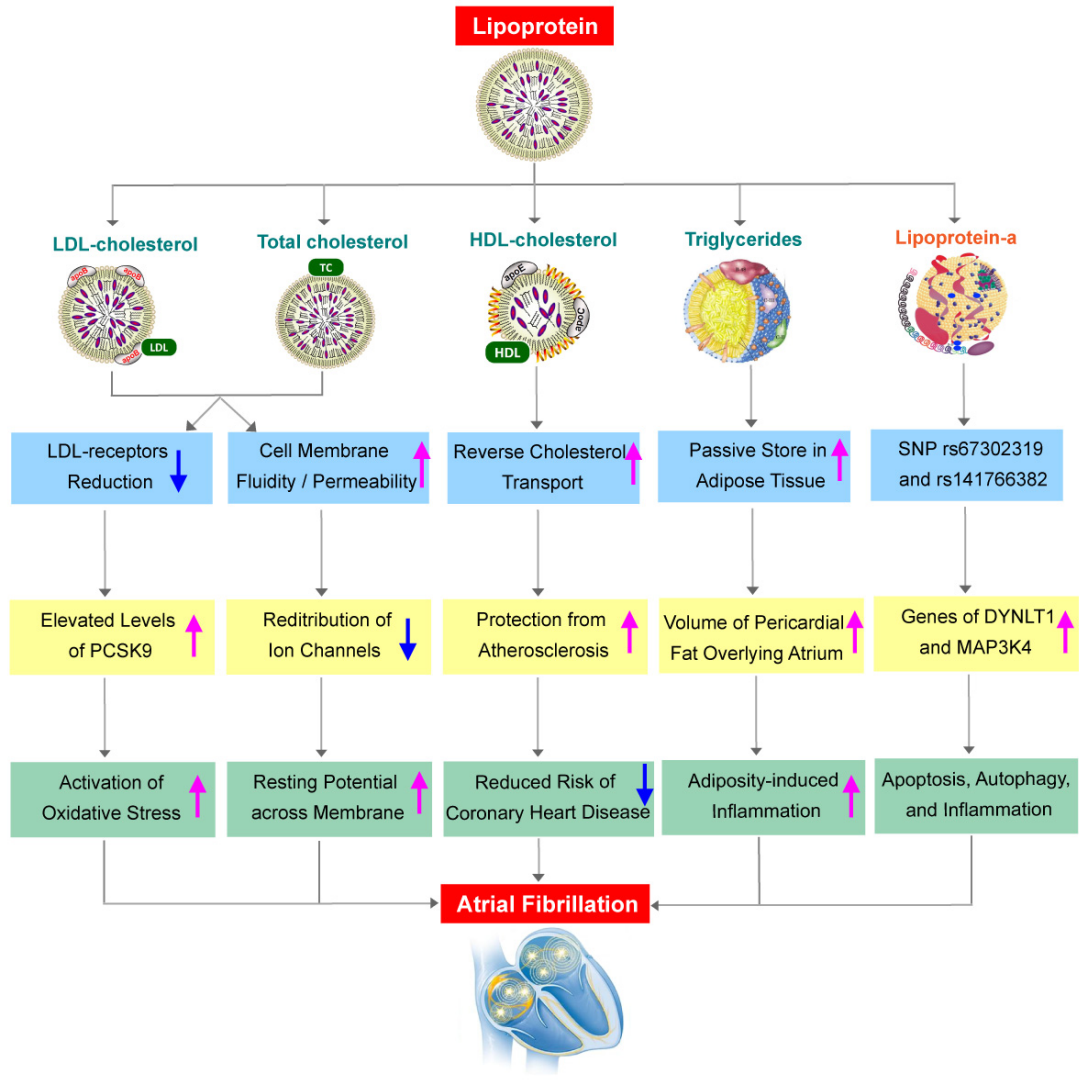
**Overview of lipid profile in atrial fibrillation and the underlying mechanisms**. Overview of lipid profile in atrial fibrillation and the underlying mechanisms. LDL, low-density lipoproteins; HDL, high-density lipoproteins; PCSK9, proprotein convertase subtilisin/kexin type 9; SNP, single nucleotide polymorphism; DYNLT1, dynein light chain type 1; MAP3K4, mitogen-activated protein kinase kinase 4.

Previous studies have shown the mechanisms behind the contrary association between lipid profiles and AF, however we are still unclear about the biological signaling (Fig. 1). The first proposed mechanism was based on the fluidity and permeability of cell membrane properties, which were influenced by cholesterol levels [47]. The levels of lipid could increase the fluidity of cell membrane, shift the allocation of ion channels, and affect the resting transmembrane potential *in vitro * [48, 49]. Taken together, the effects of lipid on membrane properties may increase the risk of AF.

It is necessary to investigate the effect of oxidative stress on the relationship between AF and lipid components in a future study. Oxidative stress is widely perceived as promoting AF development with age increasing simultaneously [50]. Oxidative stress leads to the up-regulation expression of proprotein convertase subtilisin/kexin type 9 (PCSK9) via the role of LDL-receptors [51]. Given the increasing levels of PCSK9, LDL-receptors can be reduced subsequently and then the expression of LDL-C was elevated [52]. AF patients with higher PCSK9 were more susceptible to cardiovascular events [51]. Meanwhile, age [53], inflammatory pathways [54], and hyperthyroidism [55] were related to lower expressions of TC and LDL-C and increased risk of incident AF.

### 3.2 HDL-C and TG

#### 3.2.1 Epidemiology

Prior studies have shown that the incidence rate of AF had an inverse association or no association with HDL-C or TG levels (Table 1). The dissimilarity of previous findings persists in the relationship between HDL-C or TG and incident AF. In the LIPIDOGRAM2015 cohort, the incidence rate of AF was negatively related to HDL-C (0.58 (0.46, 0.74)), but this trend was not applicable to individuals aged 75 years and older [29]. A retrospective study in Phoenix Veterans Affair Medical Center had demonstrated that among patients with diabetes there was the strongest association between low HDL levels for <31 mg/dL and highest incidence rate of AF (OR = 3.72, 95% CI 2.55–5.44, *p *< 0.05), while for patients without diabetes the trend persisted (OR = 3.69, 95% CI 2.85–4.71, *p *< 0.05) [33].

Among individuals over 75, the incidence rate of AF was negatively correlated with LDL-C/HDL-C ratio (RR = 0.75, 95% CI 0.61–0.94) [29]. A Chinese case-control study of 3469 patients revealed that compared with the lowest LDL-C/HDL-C quartile, the occurrence risk of ischemic stroke (IS) was 16.23-fold that of highest quartile in patients with non-valvular AF (NVAF) [56]. As with the Multi-Ethnic Study of Atherosclerosis (MESA) and the Framingham Heart Study, compared with HDL-C <40 mg/dL, high levels of HDL-C for ≥60 mg/dL were related to lower risk of AF (adjusted HR 0.64, 95% CI 0.48–0.87), while higher TG levels was correlated with higher AF risk in those with levels ≥200 mg/dL versus <150 mg/dL (adjusted HR 1.60, 95% CI 1.25–2.05) [34]. A meta-analysis showed that there was a negative association between HDL-C levels and AF risk (RR = 0.86, 95% CI 0.76–0.97), but TG level had no significant relationship with incident AF (RR = 1.02, 95% CI 0.90–1.17) [35].

#### 3.2.2 Pathophysiology

Based on currently present evidence, the findings contradict published research on the relationships between HDL-C or TG and AF (Fig. 1). HDL-C, unlike the other lipid components, has a protective effect on coronary atherosclerotic heart disease. As a large-scale observational study reported 45 years ago, the Framingham Heart Study first proposed that HDL-C levels had a negative correlation with CHD [57]. A recently cross-sectional study showed that HDL-C was inversely related to CHD by stimulating reverse cholesterol transport from macrophages [58]. As is well known, CHD contributed to the incidence of AF [59]. There were similarities in the negative effects of HDL-C on AF and other cardiovascular outcomes. Interestingly, it was still unclear that both HDL-C and LDL-C were negatively correlated with AF; however, the opposite correlation with other cardiovascular events. The passive store of TG increased the volume of the pericardial fat in the heart, especially overlying the atrium. Pericardial fat can induce inflammation cytokine and interact with atrial cardiomyocytes, suggesting a direct proarrhythmic effect [60]. Inflammation is a mediator between pericardial fat and AF. Therefore, adiposity-induced inflammation has had a positive effect on promoting the incidence rate of AF [60].

### 3.3 Lipoprotein-a

#### 3.3.1 Epidemiology

Previous studies have shown that there were no significant effect or protective effect of Lp(a) on AF incidence. Nevertheless, increased level of Lp(a) were positively correlated to raised risks of left atrial thrombus and cardioembolic stroke (Table 1). Compared with patients with Lp(a) ≥50 mg/dL, those with Lp(a) <10 mg/dL did not increase the incidence rate of AF (HR 0.98; 95% CI 0.82–1.17) in the community-based Atherosclerosis Risk in Communities study cohort [36]. Elevated Lp(a) level increased by 42% relative stroke risk among individuals without AF (HR 1.42; 95% CI 1.07–1.90), but not in patients with AF (HR 1.06; 95% CI 0.70–1.61 [$P_{\text{interaction}}$ for AF = 0.25]) [36].

As with the MESA cohort, individuals with Lp(a) levels ≥30 mg/dL had a 16% reduced risk of incident AF compared with those with normal levels (adjusted HR 0.84, 95% CI 0.71–0.99; *p* = 0.035) [37].

Compared to Lp(a) <100 nmol/L, there was no significant correlation with Lp(a) levels ≥100 nmol/L in AF patients by multivariable cox proportional hazard regression (adjusted HR 0.89, 95% CI 0.35–2.28, *p* = 0.81) [38]. Elevated Lp(a) was positively related to LAA stroke etiology [adjusted OR 1.48, 95% CI 1.14–1.90, per unit log10 Lp(a) increase] and recognized age as a moderator variable of this relationship ($P_{\text{interaction}}$ = 0.031) [38]. Higher levels of Lp(a) were significantly related to left atrial thrombus by the multivariate regression analysis (34.5 ± 24.1 vs 17.9 ± 13.5 mg/dL, *p *< 0.0001). Lp(a) ≥30 mg/dL had a specificity of 89% and a sensitivity of 48% for prognosticating left atrial thrombus [61].

#### 3.3.2 Pathophysiology

Although the mechanisms for such a paradoxical association are unclear, a similar relationship with AF has been reported for LDL cholesterol [35]. Non-cholesterol effects appear to underlie this relationship, driven largely by cholesterol-poor small LDL except for the larger cholesterol-rich LDL particles [62]. In a large genomic analysis, gene-specific scores for LDL cholesterol levels were not associated with AF [63]. Considering Lp(a) composition includes up to 45% cholesterol by mass and is reported as part of the LDL-cholesterol laboratory measurement, these observations could be applicable to the findings reported here. The single nucleotide polymorphism (SNP) rs67302319 and rs141766382 in Lp(a) could mediate the progress of inflammation apoptosis, and autophagy in AF pathogenesis [64]. Dynein light chain type 1 (DYNLT1), as the SNP-rs67302319 associated gene, produces apoptosis via increasing the levels of Caspase-3 and Caspase-9 [65]. Mitogen-activated protein kinase, kinase 4 (MAP3K4) is the SNP associated gene for rs141766382 and regulates the expression of interleukin-6 and interleukin-1β, which induces inflammatory reactions [66].

Elevated Lp(a) levels are forcefully related to left atrial thrombus. Lp(a) decreased the conversion progress of plasminogen to plasmin and inhibited the development of fibrinolysis, involving competing with plasminogen for comminating to endothelial and mononuclear cells to platelets [67, 68]. Lp(a) further influences the activation of plasminogen on the thrombus surface via restraining the binding function of plasminogen and tissue-type plasminogen activator to fibrin [69] (Fig. 1).

## 4. Perspectives Regarding the Relations of Lipid Profile to AF 

Although previous studies indicated a possible link of lipid disorder with AF, there are a lot of issues that need to be further addressed. First, the existing evidence on the cholesterol paradox are statistically restructured from nonmatched cohorts. Randomized controlled trials should be performed to explore the causal effects of lipids on AF risk. Moreover, there is a clear lack of follow-up data in the enrolled studies, which is essential to assess the associations between lipids and the occurrence and maintaining of AF. Finally, future research is necessary to evaluate the effect of plasma lipids on AF etiopathogenesis to guide potential clinical therapeutics. It is meaningful that the controlled blood lipid levels in AF individuals might benefit from the AF complications, in addition to quality of life.

Dyslipidemia and AF have been both prevalent in epidemic proportions around the world. These disorders may be potentially linked, and the risk of AF is fluctuating in different plasma lipid levels. However, in common with other confirmative cardiovascular disease, a strong cholesterol paradox in AF has been reported in many previous studies and large meta-analyses, suggesting that hypolipidemia with AF seem to have a better prognosis than do the hyperlipidemia with AF. Recent evidence suggests that lowering plasma levels of LDL-C, TC, HDL-C, and Lp(a) appear to be correlated with the primary prevention of AF, other than TG. Nevertheless, LDL-C, LDL-C/HDL-C, and Lp(a) have refined stroke stratification in patients with AF and were associated with a higher prevalence of left atrial thrombus and ischemic stroke. Future studies are needed to assess the impacts of LDL-C, TC, HDL-C, TG, and Lp(a) on the prevention of AF, including thrombosis and stroke risk.

## 5. Conclusions

Dyslipidemia has an important role in the risk of incident AF. The potential lipid-related mechanisms of AF development are critically affected by cell membrane properties, LDL-receptors reduction, reverse cholesterol transport, adiposity-induced inflammation, apoptosis, and autophagy. It is necessary for understanding the association between lipid profile and AF, contributing to the optimization of therapeutic strategy for the prevention of AF.
